# Special collection to commemorate 40 years of antimicrobial efflux

**DOI:** 10.1099/mic.0.001466

**Published:** 2024-06-17

**Authors:** Ayush Kumar, Jessica M. A. Blair

**Affiliations:** 1 University of Manitoba, Winnipeg, Canada; 2 Institute of Microbiology and Infection, University of Birmingham, Birmingham, UK

To mark the 40 year anniversary of the seminal description of antibiotic efflux pumps in bacteria [1] and the 30 year milestone of the characterization of the resistance-nodulation-division (RND) family of efflux pumps [2,3], we introduce this special collection on ‘Antimicrobial Efflux’[4]. Efflux pumps have long been recognized as key players in both acquired and intrinsic bacterial resistance to antibiotics and other antimicrobial agents. Over the years, our understanding of these pumps has deepened, revealing their intricate mechanisms, regulatory networks, and diverse physiological roles beyond drug resistance.

The collection of articles presented reflect the breadth and depth of research in the field of bacterial efflux pumps. From investigations into the prevalence and function of efflux pumps in diverse environmental niches, to studies elucidating the structural and regulatory aspects of specific efflux systems, to reviews examining the potential of efflux pumps as targets for therapeutic intervention, the contributions assembled here offer a comprehensive overview of the current state of knowledge and the challenges ahead.

Brown *et al*. explore the presence of multidrug efflux pumps in bacteria inhabiting oil and gas environments, shedding light on their role in biocide tolerance and pipeline asset protection [5]. Meanwhile, a review by Ayala *et al*. [6] delves into the transcriptional control of the RND efflux pump MtrCDE in *Neisseria gonorrhoeae*, underscoring its impact on antibiotic susceptibility and virulence of this important pathogen. Transcriptional regulation of RND pumps across pathogens tends to be controlled by a complex molecular network, which is often challenging to decipher. Holden *et al*. show the advantage of innovative approaches such as the TraDIS-*Xpress* transposon mutagenesis system to understand regulatory pathways in *Escherichia coli* and *Salmonella* Typhimurium [7].

The role of RND efflux pumps in the antibiotic resistance of Gram-negative organisms: *Salmonella enterica*, *E. coli* and *Pseudomonas aeruginosa* is discussed by Yamasaki *et al*. [8]. Pugh *et al*.[9] show that the complement of functional efflux pumps can vary from strain to strain and thus the studies using lab strains may not truly reflect the role of efflux pumps in strains from clinic or environment. Beyond bacterial pathogens, Islam *et al*. show that mitochondrial pumps in fungal pathogens may protect mitochondrial ribosomes from antibiotics [10].

Efflux pump interplay and its implications for antimicrobial resistance are investigated by Goetz *et al*. [11] who examine the synergistic effects of different efflux pump combinations on drug resistance in *E. coli*. Pusphker *et al*. [12] identify additional proteins involved in the translocation by the EmrE efflux pump, highlighting the complex molecular mechanisms that control the activity of these pumps and thus expand our understanding of its physiological roles beyond drug resistance.

Efflux pumps not only contribute to antibiotic resistance but also play crucial roles in bacterial adaptation, stress response, and biofilm formation. Wand and Sutton discuss the involvement of efflux pumps in increased tolerance to biocides, highlighting their impact on infection control measures as well as underscoring the role of efflux pumps in biocides and antibiotics cross-resistance [13]. Jabbari explores the application of mathematical modelling in understanding microbial efflux [14], emphasizing the utility of non-traditional approaches to study the activity of efflux pumps. Similarly, Athar *et al*., in their review, highlight the value of computer-based *in silico* research in studying the structure and function of, as they describe, *these beautifully complex machineries* [15]

Structural and functional aspects of MacAB-TolC ABC transporter are examined by Okada and Murakami [16]. This review provides valuable insights into the mechanisms of substrate recognition, transport, and inhibition of tripartite type VII ABC transporters.

Efflux pumps continue to pose significant challenges in the fight against antibiotic resistance, with the emergence of multidrug-resistant pathogens presenting a pressing global health threat. Gaurav *et al*. [17] discuss the development of efflux pump inhibitors as a promising approach to combat resistance, highlighting the potential of natural products and machine learning in the search for new pump inhibitors. The review by Wilhelm and Pos [18], however, cautions us when designing inhibitors of efflux pumps. Although the overall architecture of the RND complex is largely similar across Gram-negative bacterial species, using structural and functional data they demonstrate that there are potentially significant differences in the determinants of substrate specificity among various pumps. These differences could impede the effectiveness of a common inhibitor in disrupting their activities.

Lastly, Naidu *et al*. describe a plasmid-borne MFS family efflux pump, AadT, in *Acinetobacter baumannii* [19]*,* that may be regulated by the AdeRS two-component regulator of the RND pump AdeAB(C). This study shows that common regulatory elements can control the expression of efflux pumps belonging to different families.

In conclusion, this collection underscores the multifaceted nature of bacterial efflux pumps and their central role in antimicrobial resistance. It also highlights the progress that has been made in the last 40 years in understanding the structure, function and power of these fascinating microbial machines. We hope that the insights and findings presented here will lead to continued research efforts and collaborations aimed at tackling this critical challenge in managing the problem of antibiotic resistance.

In closing, we would like to thank all the authors and reviewers who have made this special collection possible.
